# Toward the elimination of NTDs: application of cost-effective and sensitive molecular environmental surveillance tools—a pilot study

**DOI:** 10.3389/fpara.2024.1340161

**Published:** 2024-03-26

**Authors:** Juliet Hodgson, Gideon Twieku, Gerard Quarcoo, Emmanuel Armah, Mike Yaw Osei-Atweneboana, Samuel Armoo

**Affiliations:** ^1^ Biomedical and Public Health Research Unit, Council for Scientific and Industrial Research -Water Research Institute, Accra, Ghana; ^2^ Department of Molecular Medicine, Kwame Nkrumah University of Science and Technology, Kumasi, Ghana

**Keywords:** molecular detection, urban farms, wastewater, environmental surveillance, soil-transmitted helminths

## Abstract

Neglected tropical diseases (NTDs) affect over a billion people worldwide. The 2021–2030 NTD road map calls for innovative and highly efficient interventions to eliminate or significantly reduce the burden of NTDs. These include sensitive and cost-effective diagnostic techniques for disease surveillance. Environmental surveillance has been employed effectively in this regard to measure and track infectious diseases such as polio on a population-wide scale. In this study, environmental surveillance was used as a cost-effective tool for the detection of soil-transmitted helminths (STHs) in Accra, Ghana, in an area that is populated by urban vegetable farmers. The activities of urban farmers expose them to the risk of STH infection, as well as impact the transmission in urban areas since leafy vegetables could carry infective stages of STHs. A total of 32 wastewater samples were collected from eight points on the Nima Creek (the main source of irrigation for the farmers) over a 7-week period. Real-time PCR and melt peak analysis were used to screen four STHs (i.e., *Ascaris lumbricoides*, *Necator americanus*, *Ancylostoma duodenale*, and *Trichuris trichiura*). This study revealed that *A. lumbricoides* (17 out of 32 wastewater samples, 53.3%) was the most prevalent STH, followed by *A. duodenale* (31.2%), *T. trichiura* (21.9%), and *N. americanus* (12.5%). Environmental surveillance helps in the detection of the types of STH pathogens circulating within the community and in the design of mass drug administration (MDA) strategies. This surveillance technique can also provide preliminary information for environmental modifications to help reduce STH transmission in line with the One Health approach recommended in the 2021–2030 NTD road map.

## Introduction

Neglected tropical diseases (NTDs) are a set of infectious diseases that affect approximately 1.7 billion people worldwide (Elphick-Pooley et al., 2022), mostly deprived populations in tropical regions (The Lancet Regional Health Western Pacific, 2022). Despite being preventable and treatable, NTDs cause an estimated 47.9 million disability-adjusted life years (DALYs) worldwide, resulting in significant disruptions on the social and economic well-being of infected individuals (Aya Pastrana et al., 2020). The elimination of NTDs will help achieve target 3.3 of the Sustainable Development Goals of the United Nations (Smith and Taylor, 2016).

Soil-transmitted helminths (STHs) are the most prevalent NTDs and among the most common infectious diseases in the world. They affect approximately 1.5 billion people worldwide (compared to 1.7 billion for all NTDs), causing 36.8 million DALYs (Alexander and Blackburn, 2019; World Health Organization, 2023). STHs comprise a group of intestinal nematodes that mainly include *Ascaris lumbricoides*, *Trichuris trichiura*, *Strongyloides stercoralis*, and the hookworm species, i.e., *Necator americanus* and *Ancylostoma duodenale* (World Health Organization, 2023). The clinical manifestations of STHs include abdominal pain, diarrhea, intestinal obstruction, and sometimes death by *A. lumbricoides* (Villamizar et al., 1996). *T. trichiura* and *S. stercoralis* infections can cause abdominal pain, diarrhea, and rectal prolapse (Stephenson et al., 2000). Hookworms cause anemia, fatigue, impaired cognitive development, and sometimes death (Loukas et al., 2016).

The transmission of STHs is aided by poor water, sanitation, and hygiene (WASH) practices (World Health Organization, 2023). For *A. lumbricoides* and *T. trichiura*, infection occurs through the ingestion of eggs in contaminated food, water, or on hands. For hookworms, transmission is through the penetration of the skin by larvae present in contaminated soil. Children and pregnant women are at the highest risk of associated STH morbidity (World Health Organization, 2023).

It is estimated that around 10% of people living in cities in West Africa (including Accra) are involved in urban farming (Amoah et al., 2016). The majority of these urban farmers use wastewater for irrigation (Amoah et al., 2016), exposing them to a high risk of STH infection. It is worth noting that approximately 50%–90% of the vegetables consumed in urban areas are irrigated with wastewater (Amoah et al., 2016). Since these vegetables (particularly leafy vegetables such as lettuce and spring onions) are consumed fresh, they can be a source of the transmission of STHs in cities (Pratama et al., 2023).

The primary intervention strategy for the control of STH in endemic settings is mass drug administration (MDA) with albendazole (World Health Organization, 2023). Prior to MDA, assessment of the treatment needs at the community level is performed, which mainly uses the traditional Kato-Katz diagnostic technique. Despite the many advantages of this technique, it is relatively time-consuming and requires highly skilled technicians for microscopy (Armoo et al., 2020). In line with the key recommendations of the 2021–2030 road map for NTDs, we explored the use of molecular environmental surveillance as a rapid and cost-effective technique for providing early warning data on STHs. When applied well, environmental surveillance can provide preliminary data on the impact of MDAs in endemic communities.

Wastewater surveillance involves the measurement of pathogens and substances excreted by humans (Sims and Kasprzyk-Hordern, 2020). The measurement of the microbial DNA/RNA in wastewater can provide indications of the presence of infection in a population, and the strength of the signal can be used for preliminary investigations of potential disease hotspots (Zhang et al., 2021). In Ghana, a vast majority of wastewater is expended into drains and nearby water bodies and is reused for irrigation by farmers, increasing the risk of transmission of pathogens to consumers (Amoah et al., 2016). The reuse of wastewater in farming has been to encouraged as a means to sustainable development in developing countries as it improves the farmers’ livelihood and cuts down costs. This study aimed to assess the use of wastewater surveillance as a cost-effective and sensitive environmental surveillance tool for the diagnosis of STHs in a bid to provide new insights into the diagnosis of NTDs as elimination efforts are implemented toward the 2021–2030 goals. It can be a good method for monitoring communities in terms of the reemergence of diseases following interventions.

## Materials and methods

### Ethics

Ethical clearance (CHRPE/AP/027/21) was obtained from the Committee on Human Research, Publication and Ethics of the Kwame Nkrumah University of Science and Technology (CHRPE-KNUST).

### Sampling

The study was conducted along the Nima Creek in the Greater Accra region of Ghana (5°35.5–5°35.9 N and 0°10.9–0°11.4 W). This creek has a catchment area of 6.7 km^2^ and runs along different urban settlement types in the outskirts of Accra (Mohammed et al., 2021). The upstream part of the creek is characterized by fast urbanization with residential and office accommodations, with the midstream part being surrounded by urban farming sites (Baa-Poku et al., 2013). This creek serves as the main irrigation water source for urban farmers dotted along the catchment area. There are approximately 30 registered vegetable farmers who are involved in year-round farming. The farmers cultivate vegetables such as green pepper, spring onions, lettuce, and cabbage.

**Figure 1:**
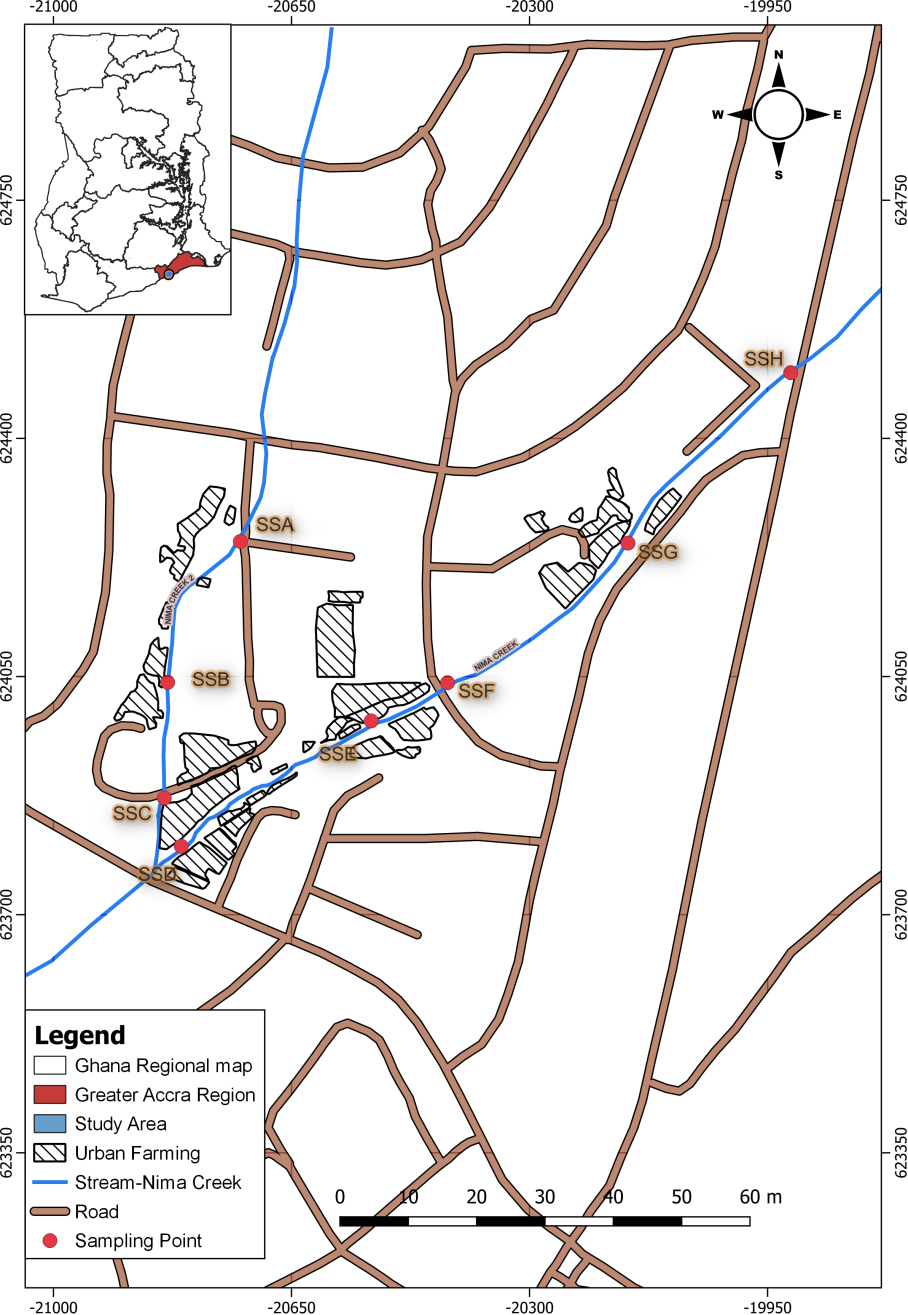
Map highlighting the sampling points along Nima Creek.

Sampling was carried out at eight sites along the Nima Creek, with IDs SSA–SSH (**Figure 1**). For sample collection, a 500-mL labeled sterile bottle was filled with wastewater from each sampling point every fortnight over an 8-week period, resulting in a total of 32 samples. The samples were kept on ice and transferred to the Biomedical and Public Health Research Unit of CSIR-Water Research Institute for processing and analysis.

### Sample processing and DNA extraction

For sample concentration, a cost-effective membrane filtration method was used. A 30-μm polycarbonate membrane filter (Sterlitech, Auburn, WA, USA) was fitted into a membrane filter holder and attached to a 15-mL syringe. A 100-mL aliquot of each sample was filtered using the membrane filter setup. The membrane (with filtrate) was then transferred into a sterile 1.5-mL microcentrifuge tube. To this, 800 µL of 2% polyvinylpyrrolidone (PVP)/phosphate-buffered saline (PBS) was added, followed by bead beating using a MagnaLyser (Roche, Basel, Switzerland) at 3,000 rpm for 1 min. DNA was extracted from 500 μL of the filtrate solution using the ZYMO Quick-DNA Kit (Zymo Research, Irvine, CA, USA) according to the manufacturer’s protocol. Prior to the DNA extraction process, phocine herpesvirus (PhHV) was added to the dislodged pellet solution and served as a positive extraction control.

### Real-time PCR and melt analysis

Screening for the presence of the four STH pathogens (i.e., *A. lumbricoides*, *N. americanus*, *A. duodenale*, and *T. trichiura*) in environmental samples was performed using the primers presented in **Table 1**. All of the real-time PCR assays (Cunningham et al., 2018) were performed using the SsoAdvanced Universal SYBR Green Supermix (2×) from Bio-Rad (Hercules, CA, USA). Each PCR reaction (total volume of 10 μL) contained 5 μL of the Supermix, 0.2 μM of each primer, 2 μL each of template DNA, and water. All real-time PCR runs were performed on the CFX96 Touch Real-Time PCR Detection System from Bio-Rad (Hercules, CA, USA). Each quantitative PCR (qPCR) run consisted of the following cycle: an initial denaturation step at 95°C for 3 min followed by 45 cycles of 95°C for 15 s, 60°C for 15 s, and 72°C for 15 s. Post-PCR melt analysis tests were conducted for all runs using the following thermal conditions: from 65°C to 95°C with a 0.2°C hold at 5 s for a plate read.

**Table 1:** Primers used in the study along with the amplicon sizes and melt temperatures.

Target	Primer name	Primer sequence	Amplicon size (bp)	Melt temp. (°C)
*A. lumbricoides*	Alum96 F	GTAATAGCAGTCGGCGGTTTCTT	88	81.00
	Alum183R	GCCCAACATGCCACCTATTC		
*N. americanus*	Na58F	CTGTTTGTCGAACGGTACTTGC	101	81.00
	Na158R	ATAACAGCGTGCACATGTTGC		
*Ancylostoma* spp.	Ad125F	GAATGACAGCAAACTCGTTGTTG	71	84.00
	Ad195R	ATACTAGCCACTGCCGAAACGT		
*T. trichiura*	18sF	TTGAAACGACTTGCTCATCAACTT	76	76.50
	18sR	CTGATTCTCCGTTAACCGTTGTC		
Phocine herpesvirus (PhHV)	PhHV-267F	GGGCGAATCACAGATTGAATC	69	80.50
	PhHV-337R	GCGGTTCCAAACGTACCAA		

In this study, a sample was viable only after the amplification of the PhHV extraction control. Each batch of PCR runs included negative no template controls. A negative result was expected for the no template control for each batch of PCR runs to qualify for further analysis. A *C*_t_ cutoff value of 40 was set. For the melt analysis, each pathogen assayed here had clinical positive samples, which were run together with the unknown wastewater samples. Confirmation of positivity for STH was based on melt peak clustering with known clinical positive controls after the melt analysis.

## Results

**Figure 2:**
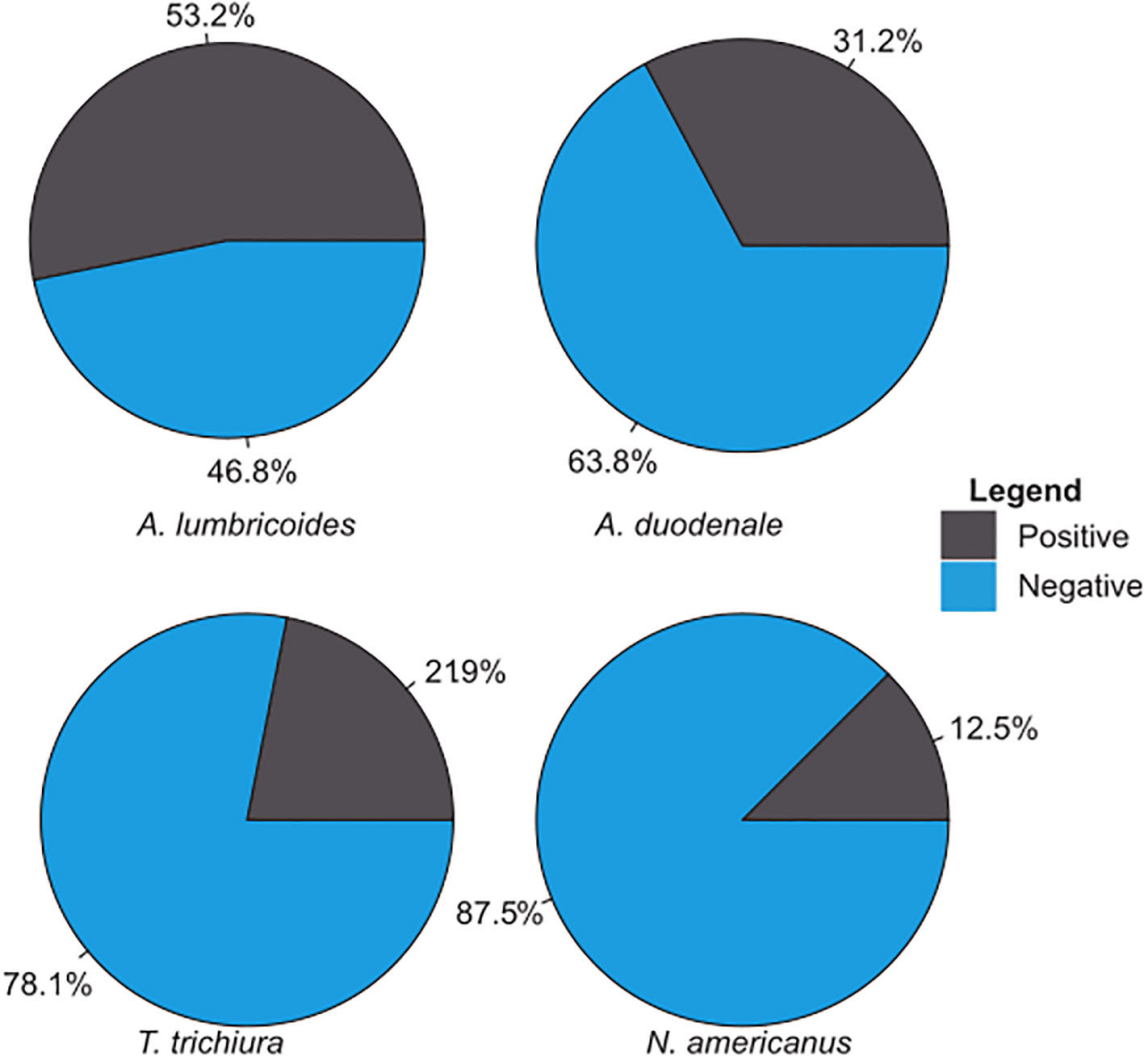
Overall prevalence of soil-transmitted helminths (STHs) from 32 environmental samples.

**Figure 3:**
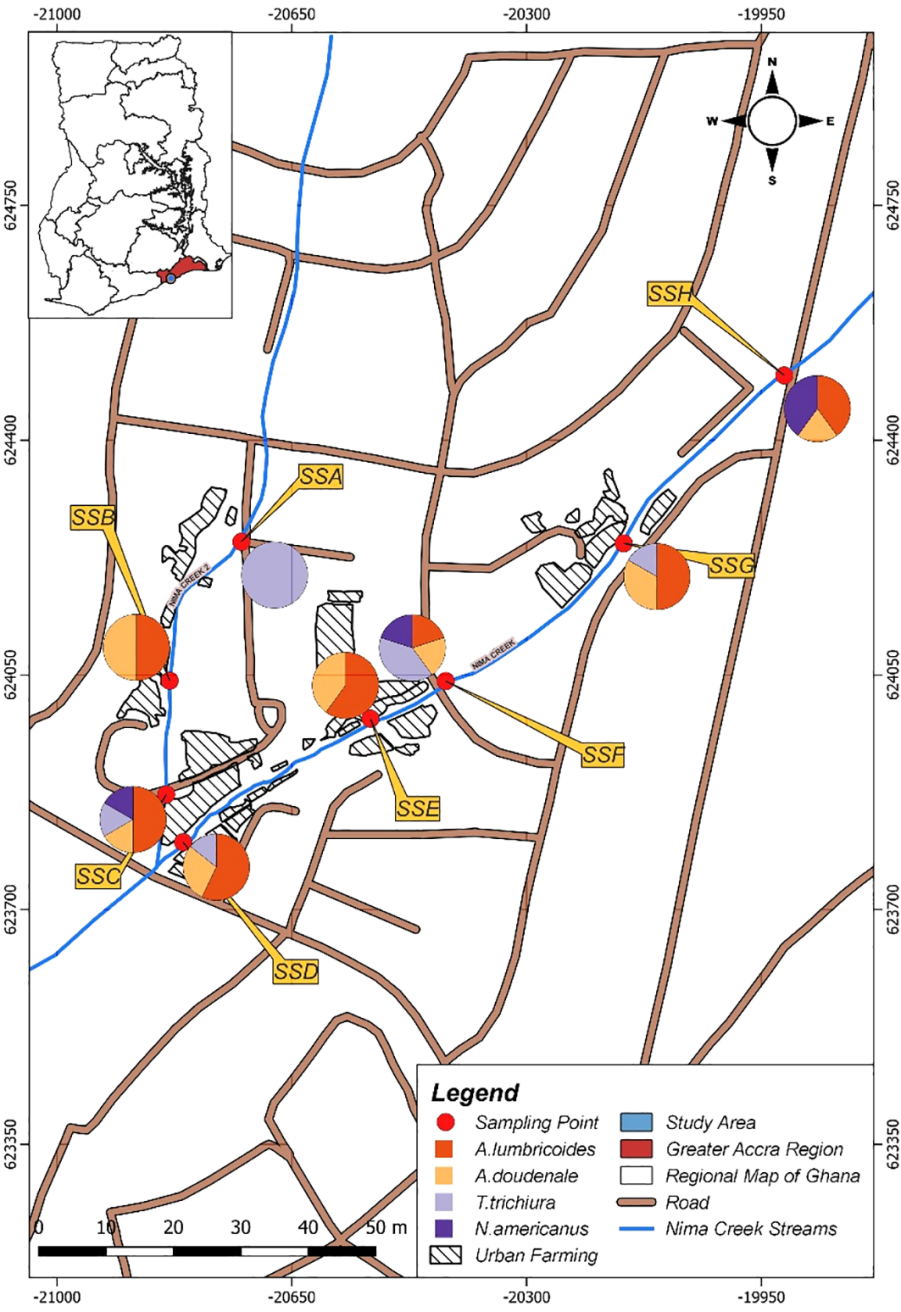
Distribution of soil-transmitted helminths (STHs) across the sampling sites.

A total of 32 environmental samples were collected from the eight study sites in an urban subsistence farming enclave in Accra, Ghana (**Figure 1**). For all samples, the amplification of the PhHV extraction control was the key quality control criterion. Each sample was screened for the presence of the four selected STHs (i.e., *A. lumbricoides*, *A. duodenale, N. americanus*, and *T. trichiura*) using a qPCR assay followed by melt analysis tests. Overall, the most common STH was *A. lumbricoides*, which was detected in 17 of the 32 samples (53.2%) (**Figure 2**). *A. duodenale* was the second most common STH, having been detected in 31.2% of the samples (**Figure 2**). This was followed by *T. trichiura* and *N. americanus*, which were detected in 21.9% and 12.5% (**Figure 2**) of the samples, respectively. Of the 32 samples, 9 (28.1%) tested positive for all four STHs, 3 (9.4%) tested positive for three STHs, 9 (28.1%) tested positive for two STHs, and 11 (34.4%) tested positive for a single STH (**Figure 3**). More details on the positivity rates at each site are presented in **Supplementary File 3**.

All eight sampling sites harbored at least one of the four STHs under investigation (**Figure 3**). Two sampling sites (SSC and SSF) harbored all four STHs (**Figure 3**). Both SSC and SSF are downstream of the Nima Creek (**Figure 1**). At site SSA, which is upstream, only *T. trichiura* was detected. Both *A. lumbricoides* and *A. duodenale* were detected at all sampling sites, except for SSA (**Figure 3**).

During baseline sampling (week 1), only two of the four pathogens—*A. lumbricoides* (one positive) and *T. trichiura* (two positives)—were detected. In each of the follow-up periods (week 3 to week 7), all four STH species were detected. The highest number of pathogen count observed throughout the study was recorded in week 7, where a total of 16 pathogens were detected. Of these, six were *A. lumbricoides*, seven were *A. duodenale*, two were *T. trichiura*, and one was *N. americanus.* A total of 9 positive samples were detected during week 3: five *A. lumbricoides*, two *A. duodenale*, one *T. trichiura*, and one *N. americanus*. Week 5 also detected a total of nine pathogen counts: five *A. lumbricoides*, one *A. duodenale*, two *T. trichiura*, and one *N. americanus* (**Figure 3**).

## Discussion

In all, *A. lumbricoides* was the most common STH detected in the wastewater samples, which is consistent with findings from studies using both environmental (Cunningham et al., 2018; Manuel et al., 2023; kinfu Gurmassa et al., 2023) and clinical samples (Cunningham et al., 2020; Chelkeba et al., 2022; Lebu et al., 2023). The differences in the prevalence rates at the different sampling sites, as shown in **Figure 3**, indicate the potential of this wastewater surveillance technique in providing preliminary information on the distribution of STH in densely populated urban communities. Although environmental surveillance data cannot point to a specific element, it has the potential to serve as an important early warning system to inform follow-up decisions for hotspot regions (Wang et al., 2022; Tiwari et al., 2023). This approach can help detect STHs circulating in the community, therefore helping to inform decisions regarding MDA as MDA programs are delivered at the community level. This is in addition to being effective in low-prevalence or near post-elimination settings.

Moreover, in this study, the differences between the weeks of sampling and the general prevalence of STHs were determined. When explored, environmental surveillance can provide preliminary information on seasonal variations in the prevalence of STHs (Oyewole and Simon-Oke, 2022). The environment plays a critical role in STH transmission since the infective stages are carried or hosted in the environment. A hostile environment, due to several conditions including weather changes, will impact the transmission of STHs. Detection of the peak STH transmission seasons will help inform interventions such as education, MDA, and modifications in the environment. This fits into the One Health approach being recommended in the 2021–2030 NTD road map.

The use of wastewater for irrigation poses a significant threat to the fight against the spread of infections such as STHs, bacteria, and protozoa (Quarcoo et al., 2022; Pratama et al., 2023). STH eggs have been detected in fresh leafy vegetables sold on the market (Pratama et al., 2023). The sampling sites in this study (**Figure 1**) are populated by urban farmers; thereby, the high prevalence of STHs detected here can have implications on the spread of STH infection in Accra. Urban farmers should be a special target group for interventions against STHs in urban communities. Apart from MDA strategies specifically tailored for these urban farmers, there should be education and behavior change to help combat the spread of STH and meet the targets of the 2021–2030 NTD road map.

In this study, we mainly focused on STHs. However, the methods described here can also be used for the detection of other pathogens, notably parasitic protozoa and bacterial and viral pathogens of public health significance (Sims and Kasprzyk-Hordern, 2020; Zhang et al., 2021). Going forward, a wider range of pathogens could be screened using probe-based qPCR to better improve the accuracy and specificity of the assays.

### Limitations

Despite the advantages and potential of wastewater surveillance, it is sometimes not possible to confirm that the parasites detected are in circulation within the sampling area. It is possible that these parasites were carried from locations outside the study area. Despite this limitation, there could be hotspot regions within a wastewater surveillance network, which can provide preliminary information for in-depth analysis that can lead to the detection of high-risk populations.

The PCR primers used here have not been previously verified for diagnosis by melt analysis. Some of the primers and samples produced nonspecific peaks. The key remedy here was that all PCR runs were accompanied by clinical positive controls. A sample was considered positive if it peaked with known clinical positives despite the presence of nonspecific peaks. It should also be noted here that a better confirmation of the positive results would be sequencing. This was not performed here due to resource limitations and is therefore an additional limitation of this study.

## Conclusion

In this study, a cost-effective and sensitive molecular diagnostic tool was used for the environmental surveillance of STHs in an area populated by urban farmers. The use of this environmental surveillance technique detected STHs in a trend similar to that found in clinical samples. Given that environmental surveillance is a less intrusive method, it could be a powerful tool for the detection of the types of STHs circulating in a community. MDAs are administered at the community level; therefore, detection of the community-level prevalence and diversity of STHs can help inform MDA intervention strategies. Variations in the prevalence and diversity of STHs at the different sampling sites and weeks indicated that environmental surveillance, when further explored, has the potential to help in the detection of STH transmission hotspot communities and seasonal variations in transmission. The environmental surveillance approach used in this study complies with the 2021–2030 NTD road map target of exploring new tools to enhance disease surveillance.

## Data availability statement

The raw data supporting the conclusions of this article will be made available by the authors, without undue reservation.

## Ethics statement

Ethical clearance (CHRPE/AP/027/21) was obtained from the Committee on Human Research, Publication and Ethics of the Kwame Nkrumah University of Science and Technology (CHRPEKNUST).

## Author contributions

JH: Formal analysis, Methodology, Visualization, Writing – original draft, Writing – review & editing. GT: Formal Analysis, Investigation, Methodology, Visualization, Writing – review & editing. GQ: Investigation, Methodology, Writing – review & editing. EA: Formal analysis, Investigation, Methodology, Writing – review & editing. MO-A: Conceptualization, Resources, Writing – review & editing. SA: Conceptualization, Investigation, Methodology, Project administration, Resources, Writing – review & editing.
