# Supplementary material for: Toward the elimination of NTDs: application of cost-effective and sensitive molecular environmental surveillance tools—a pilot study
Source: Front Parasitol. 2024 Mar 26;3:1340161. doi: 10.3389/fpara.2024.1340161 (PMC11732049; doi:10.3389/fpara.2024.1340161)
Supplement: Supplementary file 1 [file DataSheet_1.zip › Supplementary File 1.pdf]

Supplementary File 1: Coordinates of sampling sites

Sample Site ID	Latitude	Longitude
SSA	5.598788	-0.186178
SSB	5.596937	-0.187138
SSC	5.595417	-0.187183
SSD	5.594783	-0.186958
SSE	5.596430	-0.184450
SSF	5.596931	-0.183442
SSG	5.598767	-0.181058
SSH	5.601006	-0.178908
