# Supplementary material for: Toward the elimination of NTDs: application of cost-effective and sensitive molecular environmental surveillance tools—a pilot study
Source: Front Parasitol. 2024 Mar 26;3:1340161. doi: 10.3389/fpara.2024.1340161 (PMC11732049; doi:10.3389/fpara.2024.1340161)
Supplement: Supplementary file 1 [file DataSheet_1.zip › Supplementary File 3.pdf]

Supplementary File 3: Positivity rates at various sites within the sampling period

WEEK	SAMPLE ID	*A. lumbricoides*	*Ancylostoma spp*	*T. trichuris*
WEEK 1	SSA-1	NEGATIVE	NEGATIVE	NEGATIVE
	SSB-1	NEGATIVE	NEGATIVE	NEGATIVE
	SSC-1	NEGATIVE	NEGATIVE	NEGATIVE
	SSD-1	POSITIVE	NEGATIVE	NEGATIVE
	SSE-1	NEGATIVE	NEGATIVE	NEGATIVE
	SSF-1	NEGATIVE	NEGATIVE	POSITIVE
	SSG-1	NEGATIVE	NEGATIVE	POSITIVE
	SSH-1	NEGATIVE	NEGATIVE	NEGATIVE
WEEK 2	SSA-2	NEGATIVE	NEGATIVE	POSITIVE
	SSB-2	NEGATIVE	NEGATIVE	NEGATIVE
	SSC-2	POSITIVE	NEGATIVE	NEGATIVE
	SSD-2	POSITIVE	POSITIVE	NEGATIVE
	SSE-2	POSITIVE	NEGATIVE	NEGATIVE
	SSF-2	NEGATIVE	NEGATIVE	NEGATIVE
	SSG-2	POSITIVE	POSITIVE	NEGATIVE
	SSH-2	POSITIVE	NEGATIVE	NEGATIVE
WEEK 3	SSA-3	NEGATIVE	NEGATIVE	POSITIVE
	SSB-3	POSITIVE	NEGATIVE	NEGATIVE
	SSC-3	POSITIVE	NEGATIVE	NEGATIVE
	SSD-3	POSITIVE	NEGATIVE	POSITIVE
	SSE-3	POSITIVE	POSITIVE	NEGATIVE
	SSF-3	NEGATIVE	NEGATIVE	NEGATIVE
	SSG-3	POSITIVE	NEGATIVE	NEGATIVE
	SSH-3	NEGATIVE	NEGATIVE	NEGATIVE
WEEK 4	SSA-4	NEGATIVE	NEGATIVE	NEGATIVE
	SSB-4	NEGATIVE	POSITIVE	NEGATIVE
	SSC-4	POSITIVE	POSITIVE	POSITIVE
	SSD-4	POSITIVE	POSITIVE	NEGATIVE
	SSE-4	POSITIVE	POSITIVE	NEGATIVE
	SSF-4	POSITIVE	POSITIVE	POSITIVE
	SSG-4	POSITIVE	POSITIVE	NEGATIVE
	SSH-4	POSITIVE	POSITIVE	NEGATIVE
