# Supplementary figures and images for: Toward the elimination of NTDs: application of cost-effective and sensitive molecular environmental surveillance tools—a pilot study
Source: Front Parasitol. 2024 Mar 26;3:1340161. doi: 10.3389/fpara.2024.1340161 (PMC11732049; doi:10.3389/fpara.2024.1340161)

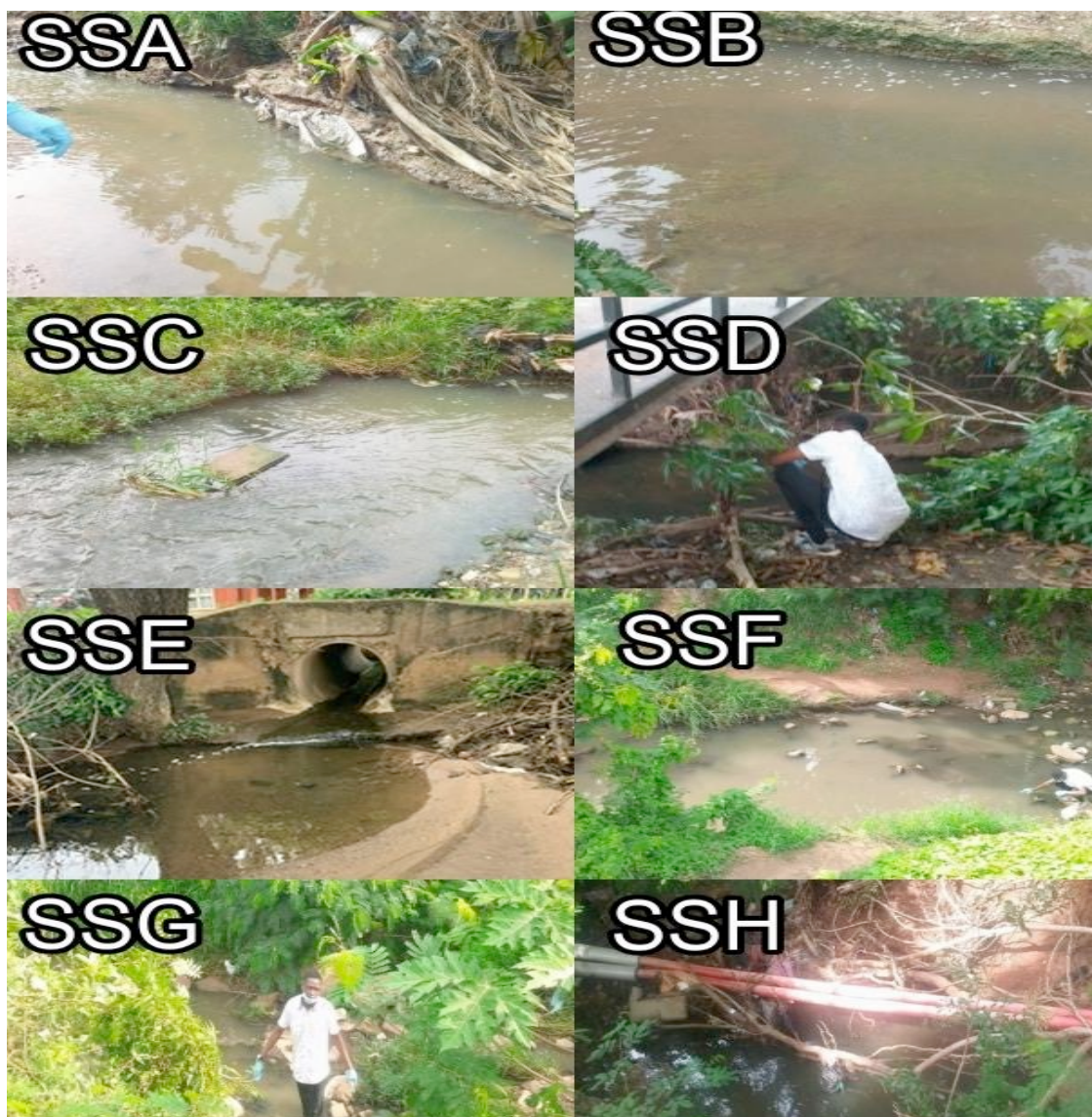

**Supplementary File 2: Pictures of sampling sites**

Supplement: Supplementary file 1 [file DataSheet_1.zip › Supplementary File 2.pdf]
